# Physical Training

**Published:** 1860-07

**Authors:** 


### PHYSICAL TRAINING.

THE following is a continuation of the article in the June number on the same subject, and merits especial attention, particularly on the part of those who wish to obtain more physical vigor, and wish to do it in a safe way, avoiding the shocks, irregularities, strainings, wrenchings, etc., which attend gymnastic trainings:

"It has been proved by experiment that every part of the firmest bones is successively absorbed and deposited. The bones and their ligaments, the muscles and their tendons, all the finer and the more flexible parts of the body, are continually renewed, and as properly a secretion as the saliva that flows from the mouth, or the moisture that bedews the surface. The health of all the parts, and their soundness of structure, depend on this perpetual absorption and perpetual renovation; and exercise, by promoting at once absorption and secretion, promotes life without hurrying it, renovates all the parts, and preserves them apt and fit for every office. When the human frame is thus capable of being altered and renovated, it is not surprising that the art of training should be carried to a degree of perfection almost incredible, and that by certain processes, the breath, (or wind,) strength, and courage of man, should be so greatly improved as to enable him to perform the most laborious undertakings. That such effects have been produced is unquestionable, being fully exemplified in the astonishing exploits of celebrated pedestrians and pugilists, which are the infallible results of such preparatory discipline. The skillful trainer attends to the state of the bowels, the lungs, and the skin; and he uses such means as will reduce the fat, and at the same time invigorate the muscular fibers. The patient is purged by drastic medicines; he is sweated by walking under a load of clothes, and by lying between feather-beds. His limbs are roughly rubbed. His diet is beef or mutton; his drink, strong ale; and he is gradually inured to exercise by repeated trials in walking and running. By exterminating the fat, emptying the cellular substance, hardening the muscular fiber, and improving the breath, a man of the ordinary frame may be made to fight for one hour, with the utmost exertion of strength and courage, or to go over one hundred miles in twenty-four hours. The most effectual process for training in the days of Tom Cribb, was that practiced by the celebrated pedestrian, Captain Barclay, and the particular method he adopted has not only been sanctioned by professional men, but met with the unqualified approbation of amateurs. The following statement, therefore, contains the most approved rules; and it is presented to the reader as the result of much experience, founded on the theoretic principles of the art, and as practiced by Captain Barclay:

"The pedestrian, who may be supposed in tolerable condition, enters upon his training with a regular course of physic, which consists of three doses. Glauber salts are generally preferred, and from one ounce and a half to two ounces are taken at each time, with an interval of four days between each dose. After having gone through the course of physic, he commences his regular exercise, which is gradually increased as he proceeds in the training. When the object in view is the accomplishment of a pedestrian match, his regular exercise may be from twenty to twenty-four miles a day. He must rise at five in the morning, run half a mile at the top of his speed up-hill, and then walk six miles at a moderate pace, coming in about seven to breakfast, which should consist of beef-steaks or mutton-chops underdone, with stale bread and old beer. After breakfast, he must again walk six miles at a moderate pace, and at twelve lie down in his bed without his clothes for half an hour. On getting up, he must walk four miles, and return by four to dinner, which should also be beef-steaks or mutton-chops, with bread and beer as at breakfast. Immediately after dinner he must resume his exercise, by running half a mile at the top of his speed, and walking six miles at a moderate pace. He takes no more exercise for that day, but retires to bed about eight, and next morning proceeds in the same manner. After having gone on in this regular course for three or four weeks, the pedestrian must take a four-mile sweat, which is produced by running four miles, in flannel, at the top of his speed. Immediately on returning, a hot liquor is prescribed, in order to promote the perspiration, of which he must drink one English pint. It is termed the sweating liquor, and is composed of the following ingredients, namely: one ounce of caraway-seed; half an ounce of coriander-seed; one ounce of root-liquorice; and half an ounce of sugar-candy; mixed with two bottles of cider, and boiled down to one half. He is then put to bed in his flannels, and being covered with six or eight pairs of blankets and a feather-bed, must remain in this state from twenty-five to thirty minutes, when he is taken out and rubbed perfectly dry. Being then well wrapped in his great coat, he walks out gently for two miles, and returns to breakfast, which, on such occasions, should consist of a roasted fowl. He afterwards proceeds with his usual exercise. These sweats are continued weekly, till within a few days of the performance of the match, or, in other words, he must undergo three or four of these operations. If the stomach of the pedestrian be foul, an emetic or two must be given about a week before the conclusion of the training, and he is now supposed to be in the highest condition.

"Besides his usual or regular exercise, a person under training ought to employ himself in the intervals in every kind of exertion which tends to activity, such as cricket, bowls, throwing quoits, etc., so that, during the whole day, both body and mind may be constantly occupied.

"The beneficial consequences, both to the body and the mind, arising from training, are not merely temporary, but may be made permanent by proper care and attention. The simplicity of the rules is a great recommendation to those who may be desirous of trying the experiment, and the whole process may be resolved into the following principles: 1st. The evacuating, which cleanses the stomach and intestines. 2d. The sweating, which takes off the superfluities of flesh and fat. 3d. The daily course of exercise, which improves the wind and strengthens the muscles; and lastly, The regimen, which nourishes and invigorates the body.

"The criterion by which it may be known whether a man be in good condition, or, what is the same thing, has been properly trained, is the state of the skin, which becomes smooth, elastic, and well-colored, or transparent. The flesh is also firm, and the person trained feels himself light and full of spirits. But in the progress of the training, his condition may be ascertained by the effect of the sweats, which cease to reduce his weight; and by the manner in which he performs one mile at the top of his speed. It is as difficult to run a mile at the top of one's speed as to walk a hundred; and, therefore, if he performs this short distance well, it may be concluded that his condition is perfect, or that he has derived all the advantages which can possibly result from the training process.

"Training for pugilism is nearly the same as for pedestrianism, the object in both being principally to obtain additional wind and strength. The process observed by Cribb, the champion of England, preparatory to his grand battle with Molineaux, which took place September 29, 1811, is as follows:

"THE TRAINING OF TOM CRIBB.

"The champion arrived at Ury, Capt. Barclay's residence, July 7 of that year. He weighed 224 lbs., and from his mode of living in London, and the confinement of a crowded city, he had become corpulent, big-bellied, full of gross humors, and short-breathed; and it was with difficulty he could walk ten miles. He first went through a course of physic, which consisted of three doses; for two weeks he walked about as he pleased, and generally traversed the woods and plantations with a fowling-piece in hand. The reports of his musket resounded every where through the groves and the hollows of that delightful place, to the great terror of the magpies and wood-pigeons. After amusing himself in this way for about a fortnight, he then commenced his regular walking exercise, which was at first about ten or twelve miles a day. It was soon after increased to eighteen or twenty; and he ran regularly, morning and evening, a quarter of a mile at the top of his speed. In consequence of his physic and exercise, his weight was reduced, in the course of five weeks, from 224 lbs. to 205 lbs. At this period he commenced his sweats, and took three during the month he remained at Ury afterwards; and his weight was gradually reduced to 187 lbs., which was ascertained to be his pitch of condition, as he could not reduce farther without weakening. During the course of his training, the champion went twice to the Highlands, and took strong exercise. He walked to Mar Lodge, which is about sixty miles distant from Ury, where he arrived to dinner on the second day, being now able to do thirty miles a day with ease, and probably he could have walked twice as far if it had been necessary. He remained in the Highlands about a week each time, and amused himself with shooting. The principal advantage which he derived from these expeditions was the severe exercise he was obliged to undergo in following Captain Barclay. He improved more in his strength and wind by his journeys to the Highlands than by any other part of the training process. His diet and drink were the same as used in the pedestrian regimen, and in other respects, the rules previously laid down were generally applied to him. That he was brought to his ultimate pitch of condition was evident from the high state of health and strength in which he appeared when he mounted the stage to contend with Molineaux, who has since confessed that, when he saw his fine condition, he totally despaired of gaining the battle. Cribb was altogether about eleven weeks under training, but he remained only nine weeks at Ury. Besides his regular exercise, he was occasionally employed in sparring at Stonehaven, where he gave lessons in the pugilistic art. He was not allowed much rest, but was constantly occupied in some active employment. He enjoyed good spirits, being all the time fully convinced that he would beat his antagonist.

He was managed, however, with great address, and the result corresponded with the wishes of his friends.

"HOW THE BENICIA BOY WAS TRAINED.

" He rises at six o'clock A.M., and strikes out a couple of hundred times with small dumb-bells just to stretch the muscles. As soon as he is dressed, he takes a walk of about three miles. Upon reaching home on his return, he does some 'sprint' running at the top of his speed—a hundred yards, say, six or seven times. In this performance, Cusick and Falkland stand at either end of the distance, with watches in their hands, giving him half a minute's time for rest at the score. He then goes into his room, and if perspiring freely, (which is seldom the case,) is rubbed down. Then, after a rest, breakfast is served precisely at eight. This meal is composed of mutton-chops or beef-steek cooked very rare. He is very particular as to the time of his meals. Half an hour after breakfast, he takes a salt-water bath. The bath is prepared every day, and consists of soft water and rock-salt. After this he puts on his sweating-suit, and at nine o'clock, accompanied by Cusick, (and now with McDonald or Cusick,) he starts out for his ten-mile walk, carrying in either hand a three-pound dumb-bell. The first four miles are covered at a gentle pace, after which he increases his speed gradually. When within four miles of home on his return, he puts on a mask (to sweat the face) made of white flannel, with openings for the mouth and eyes, but none for the nose. He then commences a pace of ten minutes to the mile, finishing the last mile home at a Flora Temple speed, always leaving the company behind. As soon as he gets into his room, he sits by the fire to assist the perspiration, and when he thinks he has had enough of it, he strips to the buff, and is briskly rubbed down. Now, during his hard training, he takes an egg in a glass of sherry after this. It is then half-past eleven, when he proceeds to the barn, and strikes out the dumb-bells, slings the clubs, or battles with the bag. Exercising in this manner for some time, he walks into farmer Pocock's library, and selects a book to amuse himself with until dinner, which is served at one o'clock precisely, consisting of roast beef, roast mutton, or boiled mutton. A rest of an hour or so intervenes, which is consumed in reading or talking, when he accouters himself for his afternoon's walk of eight or nine miles. This he commences at 2:30, or thereabouts. He returns at near five o'clock, when he changes his clothes, and then takes another rest before tea, which is at six o'clock, consisting of dry toast, no butter, weak black tea, with occasionally an egg. Half an hour or so after this, he commences his 'sprint' running. He then returns to the house, and sits up talking or reading until nine o'clock, when he takes a bowl of thin oat-meal gruel, and at ten o'clock he retires to bed, to rise again at six next morning, and go through the same course of exercise, diet, and treatment.

"SAYERS IN HIS TRAINING.

" Sayers rises from his virtuous couch at six o'clock in the morning, and soon after starts out for a quiet walk of three miles, or thereabouts, returning leisurely to his habitation. At eight o'clock breakfast is announced, for which the champion is now ready. The meal is plain, consisting, as a general thing, of mutton-chops, (with the addition, occasionally, of eggs,) with a single cup of strong tea, of the proper strength of which Sayers is allowed to be the judge. After having finished breakfast, he takes a rest of an hour or two, the time being enlivened by the telling of anecdotes by Bob Fuller, his trainer; Sayers now and then, in turn, regaling Bob with a laughable yarn. At the conclusion of this mirthful repast, the champion goes out for his long walk, which he terms the 'sweating process.' The distance covered in this pedestrian-excursion is from twelve to fourteen miles, during which the champion carries a seven-pound dumb-bell in each hand. By the time he gets through with this performance, and returns home, it is 12 o'clock. He is then rubbed dry with hard towels, next washed with cold water, and again rubbed vigorously with dry towels, which brings the blood to the surface of the skin, and gives a clear and healthy glow to the cuticle. Fuller now puts on the finishing touches like a skillful painter, and to attain this end he dons a pair of horse-hair gloves, and with these he gives the champion a rubbing such as but few persons could undergo.

"After submitting to these 'gentle and soothing' specimens of the veteran Bob's handiwork, the skin of the champion is moistened with Irish whisky, after which he is incased in a thick, dry suit of flannel, and then he saunters forth for a short ramble, returning home to dinner at two o'clock. This meal consists of roast beef, roast or boiled mutton, varied from day to day. Of course, the morning exercise has given him a keen appetite, and he discusses the fare with a lively sense of the importance of the occasion. The inner man satisfied with a due allowance of plain but wholesome food, a 'season of rest' is indulged in—the champion being permitted to use his own judgment at to the requisite length of the time for the *siesta*—at the expiration of which, another walk of nine or ten miles is prescribed, in thick clothing, which, on returning home, is immediately changed for a dry flannel suit. Thus wears the day along, and from the hour of returning from the afternoon walk, generally about five o'clock, Tom is master of his own time, and passes it, as in his judgment, may seem most fit and proper. The last meal is extremely simple, consisting of dry toast and tea, with, very frequently, a beaten egg or two in the latter. After resting sufficiently, he takes a gentle walk of four or five miles over the heath, and then returns home to entertain, or be entertained by any friends who may have called to see him. At half-past nine o'clock, 'balmy sleep, tired Nature's sweet restorer,' wooes him to her couch; when, to use the champion's own words, he enjoys 'most delicious repose in the arms of *Mons. Murphy.*'"

If for brutal honors men like these encounter self-denials with heroic resolution and cheerful acquiescence, how ought we to be willing to crucify "the flesh with the affections and lusts," in order that we might "win" the meed of immortality; might secure that high health of body and manly vigor which are essential to the safest and purest enjoyment of religion; essential to an active and efficient life of good doing, a death of ease, of triumph, and beatific Faith, in being that same "day" —"in Paradise."

Men of low nature "endure" "joyfully" the self-denials of the appetite, and the most laborious exercises, to prepare them for their brutal conflicts, and in the engagement, encounter the most fearful "millings" courageously and uncomplainingly, that they may obtain a "corruptible crown." Let us, then, who seek an "incorruptible that fadeth not away," consider habitually, that it is no trial to deny ourselves "the world, the flesh, and their blandishments," only if thereby we are able so "to fight that we may obtain."

